# The WSES/SICG/ACOI/SICUT/AcEMC/SIFIPAC guidelines for diagnosis and treatment of acute left colonic diverticulitis in the elderly

**DOI:** 10.1186/s13017-022-00408-0

**Published:** 2022-01-21

**Authors:** Paola Fugazzola, Marco Ceresoli, Federico Coccolini, Francesco Gabrielli, Alessandro Puzziello, Fabio Monzani, Bruno Amato, Gabriele Sganga, Massimo Sartelli, Francesco Menichetti, Gabriele Adolfo Puglisi, Dario Tartaglia, Paolo Carcoforo, Nicola Avenia, Yoram Kluger, Ciro Paolillo, Mauro Zago, Ari Leppäniemi, Matteo Tomasoni, Lorenzo Cobianchi, Francesca Dal Mas, Mario Improta, Ernest E. Moore, Andrew B. Peitzman, Michael Sugrue, Vanni Agnoletti, Gustavo P. Fraga, Dieter G. Weber, Dimitrios Damaskos, Fikri M. Abu-Zidan, Imtiaz Wani, Andrew W. Kirkpatrick, Manos Pikoulis, Nikolaos Pararas, Edward Tan, Richard Ten Broek, Ronald V. Maier, R. Justin Davies, Jeffry Kashuk, Vishal G. Shelat, Alain Chicom Mefire, Goran Augustin, Stefano Magnone, Elia Poiasina, Belinda De Simone, Massimo Chiarugi, Walt Biffl, Gian Luca Baiocchi, Fausto Catena, Luca Ansaloni

**Affiliations:** 1 IRCCS Policlinico San Matteo Foundation, General Surgery, Pavia, Italy; 2 General Surgery Department, School of Medicine and Surgery, Milano-Bicocca University, Monza, Italy; 3 Emergency Surgery Unit, State University of Pisa, Cisanello Hospital, Pisa, Italy; 4 Department of Surgery and Transplants, AOU San Giovanni di Dio and Ruggi d’Aragona, University of Salerno, Fisciano, Italy; 5 Geriatrics Unit, Department of Clinical and Experimental Medicine, University of Pisa, Pisa, Italy; 6 Department of Clinical Medicine and Surgery, University of Naples “Federico II”, Naples, Italy; 7 Emergency Surgery and Trauma, Fondazione Policlinico Universitario “A. Gemelli” IRCCS, Rome, Italy; 8 Department of Surgery, Macerata Hospital, Macerata, Italy; 9 Division of Infectious Diseases, Department of Clinical and Experimental Medicine, University of Pisa, Pisa, Italy; 10 Department of Surgery, S. Anna University Hospital and University of Ferrara, Ferrara, Italy; 11 Medical School, General Surgery and Surgical Specialties Unit, S. Maria University Hospital University of Perugia, Terni, Italy; 12 Division of General Surgery, Rambam Health Care Campus, Haifa, Israel; 13 Emergency Room Brescia Spedali Civili General Hospital, Brescia, Italy; 14 Department of Robotic and Emergency Surgery, Manzoni Hospital, ASST Lecco, Lecco, Italy; 15 Abdominal Center, Helsinki University Hospital and University of Helsinki, Helsinki, Finland; 16 Department of Management, Lincoln International Business School, University of Lincoln, Lincoln, UK; 17 Alma Mater Studiorum University, Bologna, Italy; 18 Department of Surgery, University of Colorado, Denver Health Medical Center, Denver, CO USA; 19 Department of Surgery, University of Pittsburgh School of Medicine, UPMC-Presbyterian, Pittsburgh, PA USA; 20 Donegal Clinical Research Academy, Emergency Surgery Outcome Project, Letterkenny University Hospital, Donegal, Ireland; 21 Intensive Care Unit, Bufalini Hospital, Cesena, Italy; 22 Surgery Department, Faculdade de Ciências Médicas (FCM), Unicamp Campinas, Campinas, SP Brazil; 23 Department of General Surgery, Royal Perth Hospital, Perth, Australia; 24 Department of Surgery, Royal Infirmary of Edinburgh, Edinburgh, UK; 25 Department of Surgery, College of Medicine and Health Sciences, UAE University, Al-Ain, United Arab Emirates; 26 Department of Minimal Access and General Surgery, Government Gousia Hospital, Sringar, Kashmir India; 27 General, Acute Care, Abdominal Wall Reconstruction, and Trauma Surgery, Foothills Medical Centre, Calgary, AB Canada; 28 3Rd Department of Surgery, Attiko Hospital, MSc “Global Health-Disaster Medicine”, National and Kapodistrian University of Athens (NKUA), Athens, Greece; 29 General Surgery, Dr Sulaiman Al Habib/Alfaisal University, Riyadh, Saudi Arabia; 30 Department of Surgery, Radboud University Medical Centre, Nijmegen, The Netherlands; 31 Department of Surgery, University of Washington, Seattle, WA USA; 32 Cambridge Colorectal Unit, Addenbrooke’s Hospital, Cambridge University Hospitals NHS Foundation Trust, Cambridge, UK; 33 Department of Surgery, Assia Medical Group, Tel Aviv University Sackler School of Medicine, Tel Aviv, Israel; 34 Department of General Surgery, Tan Tock Seng Hospital, Singapore, Singapore; 35 Faculty of Health Sciences, University of Buea, Buea, Cameroon; 36 Department of Surgery, University Hospital Centre, Zagreb, Croatia; 37 General Surgery I, ASST Papa Giovanni XXIII Hospital, Bergamo, Italy; 38 Department of General and Metabolic Surgery, Poissy and Saint Germain en Laye Hospitals, Poissy, France; 39 Trauma Surgery Department, Scripps Memorial Hospital, La Jolla, CA USA; 40 Department of General Surgery, ASST Cremona, University of Brescia, Cremona, Italy; 41 General and Emergency Surgery Department, Bufalini Hospital, AUSL Romagna, Cesena, Italy

**Keywords:** Acute diverticulitis, Elderly, Surgery in elderly

## Abstract

Acute left colonic diverticulitis (ALCD) in the elderly presents with unique epidemiological features when compared with younger patients. The clinical presentation is more nuanced in the elderly population, having higher in-hospital and postoperative mortality. Furthermore, geriatric comorbidities are a risk factor for complicated diverticulitis. Finally, elderly patients have a lower risk of recurrent episodes and, in case of recurrence, a lower probability of requiring urgent surgery than younger patients. The aim of the present work is to study age-related factors that may support a unique approach to the diagnosis and treatment of this problem in the elderly when compared with the WSES guidelines for the management of acute left-sided colonic diverticulitis. During the 1° Pisa Workshop of Acute Care & Trauma Surgery held in Pisa (Italy) in September 2019, with the collaboration of the World Society of Emergency Surgery (WSES), the Italian Society of Geriatric Surgery (SICG), the Italian Hospital Surgeons Association (ACOI), the Italian Emergency Surgery and Trauma Association (SICUT), the Academy of Emergency Medicine and Care (AcEMC) and the Italian Society of Surgical Pathophysiology (SIFIPAC), three panel members presented a number of statements developed for each of the four themes regarding the diagnosis and management of ALCD in older patients, formulated according to the GRADE approach, at a Consensus Conference where a panel of experts participated. The statements were subsequently debated, revised, and finally approved by the Consensus Conference attendees. The current paper is a summary report of the definitive guidelines statements on each of the following topics: diagnosis, management, surgical technique and antibiotic therapy.

## Background

Diverticulitis results from a microscopic or macroscopic perforation of a diverticulum due to diverticular inflammation and focal necrosis. Diverticulitis can present in about 10–25% of patients with diverticulosis and can be uncomplicated (symptomatic uncomplicated diverticular disease, SUDD) and complicated. Complicated diverticulitis could be associated with the formation of abscess, fistula, bowel obstruction, or frank perforation. Patients of Western nations are overwhelmingly likely to have left-sided diverticulosis (90% of cases), whereas those of Asian and African descent are likely to have the right-sided disease (70–74% of cases) [1]. The prevalence of acute left colonic diverticulitis (ALCD) increases with age. The lifetime prevalence of diverticular disease, extracted from the Health Search Database of Italian General Practitioners, increases from 10% among patients under 50 years old to 33% among patients between 60 and 69 years old [2]. However, in the last decade, the prevalence of hospitalization for acute diverticulitis has increased among patients under 70 years old, while remaining unchanged for patients aged over 70 years [3].

ALCD in elderly patients has different epidemiological features when compared with younger patients. The clinical presentation is more nuanced in the elderly population. In an observational study by Lizardi-Cervera focusing on patients with ALCD [4], only 50% of patients older than 65 years presented with abdominal pain in the lower quadrants, 17% had a fever and 43% did not have leucocytosis. On the contrary, a higher proportion of older patients presented with diverticular bleeding [4, 5]. According to some authors [6–8], the risk factor for complicated acute diverticulitis is not older age by itself, but the presence of comorbidities, often associated with advanced age.

The results of two meta-analyses on patients with ALCD [7, 9] showed that the risk of urgent surgery during a primary episode is similar when comparing patients younger and older than 50 years of age. However, as can be expected, the in-hospital mortality of patients admitted for ALCD is higher among older patients; in the last decade, increased in-hospital mortality was demonstrated in the patient cohort aged 70 years and older [2]. Again, it is not clear whether the age itself or the associated comorbidities influence mortality. In a large study by Sirinthornpunya [6], the multivariate analysis showed comorbidities as the only significant risk factor for in-hospital mortality.

Similar to elderly patients with severe sepsis who underwent alimentary tract surgery [10], advanced age persisted as an independent predictor of postoperative mortality after emergency surgery for ALCD. The observed mortality rates were 1.6% in patients younger than 65 years, 9.7% in patients between 65 and 79 years and 17.8% in patients above 80 years [11].

Regarding the risk of recurrence after the first episode of ALCD, according to most authors [7, 9, 12], the risk is significantly lower in older patients compared with younger ones. In a large study on patients over 67 years of age [12], the proportion of patients without recurrence was 83% and among patients over 80 years the recurrences were even less likely. The need for urgent surgery for recurrence was also significantly lower among older patients (cumulative risk 7.3% in patients younger than 50 years vs 4.3% in patients older than 50 years) [9]. Currently, there are no specific guidelines for the management of ALCD focusing on elderly patients.

The aim of the Consensus Conference held in Pisa in September 2019 was to investigate age-related factors that could influence a different approach, compared with the WSES guidelines for the management of acute left-sided colonic diverticulitis [13, 14], in terms of diagnosis and management of elderly patients with ALCD.

## Material and methods

The World Society of Emergency Surgery (WSES) along with the Italian Society of Surgical Physiopathology (SIFIPAC), the Italian Society of Geriatric Surgery (SICG), the Italian Hospital Surgeons Association (ACOI), the Italian Emergency Surgery and Trauma Association (SICUT) and the Academy of Emergency Medicine and Care (AcEMC) nominated a scientific committee for the development of guidelines for the diagnosis and the treatment of ALCD in the elderly patients.

Similar to the age cut-off established in the guidelines on appendicitis and on cholecystitis in the elderly [15], we elected to define “elderly” as patients aged over 65 years.

Four areas of interest were identified by the scientific committee: diagnostic, management, surgical technique and antibiotic therapy. Using the PICO framework, many focus questions were generated and constructed for each area of interest. An electronic bibliography search on PubMed and EMBASE was used to perform a systematic review of the existing literature. Researchers reviewed the literature and used the GRADE methodology to develop evidence-based responses to the questions grading the quality of evidence and assigning the recommendation's strength [16]. According to GRADE methodology, the quality of evidence was assessed and classified, into four levels: high, moderate, low and very low; the consequent recommendations were made based on the level of evidence and were classified into two levels: strong recommendation in favour or against; weak recommendation (suggestion) in favour or against.

During the 1° Pisa Workshop of Acute Care & Trauma Surgery in Pisa (Italy) in September 2019, each proposed statement was discussed, along with the results of the literature review, with the participation of a panel of experts, including members of WSES, SIFIPAC, SICG, ACOI, SICUT, AcEMC. Each statement was then voted upon by the audience and was approved if it reached at least 80% of votes in favour. Where there was discordance, the statement was improved with panel input until approval was granted by the assembly.

**Fig. 1:**
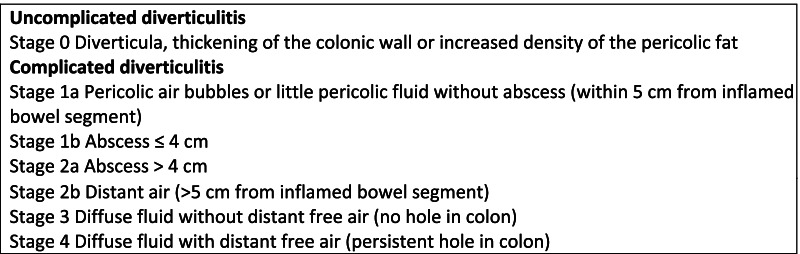
WSES left colonic diverticulitis classification [17]

Diverticulitis severity was graded according to the WSES ALCD classification (Fig. 1) [17].

## Results

### Diagnosis

1. *Could the diagnosis of acute left colonic diverticulitis be based on only clinical signs, symptoms and laboratory test in elderly patients?*

- **Statement 1.1 In the elderly population, we suggest against basing the diagnosis of acute left colonic diverticulitis on only patient clinical signs, symptoms and laboratory tests.** ***[Conditional recommendation, very low-quality evidence]***
- **Statement 1.2 We suggest that elderly patients presenting with abdominal guarding or pain in the lower left abdomen on physical examination undergo appropriate imaging for suspected diverticulitis, regardless of the value of leukocytes and of C-reactive protein (CRP).** ***[Conditional recommendation, very low-quality evidence]***

As stated in the WSES Guidelines [13, 14] in the general population, the clinical diagnosis of ALCD alone is not sufficiently accurate for patients with suspected diverticulitis. However, pain in the lower left abdomen, elevate temperature and absence of vomiting could suggest a diagnosis of ALCD [13, 14]. The reported sensitivity and specificity of the clinical diagnosis of ALCD in the general population are 0.68 and 0.98, respectively [18].

Several authors have developed clinical scoring systems in order to minimize secondary care diagnostics in suspected ALCD [19–21], but these were validated on small sample sizes composed of the general population. Bolkenstein et al. [22] developed a diagnostic prediction model distinguishing complicated from uncomplicated ALCD, validated on 475 patients presenting to the emergency department (ED) with a clinically suspected episode of diverticulitis. This model included abdominal guarding, CRP level and leucocytes count. The final model would have avoided secondary care diagnostics in 25% of all patients, with a negative predictive value (NPV) of 96%. Still, the mean age of the tested population was 61 years of age. Many studies have demonstrated that clinical manifestations are insufficient for the diagnosis of ALCD. Longstreth et al., in a retrospective electronic record-based analysis, reported that nearly 5% of patients with severe diverticulitis presented without fever or leucocytosis [23]. In the study by Kaser et al. on 247 patients [24], if a computed tomography (CT) scan was performed only with a CRP above 50 mg/l, 46 CT scan would have been avoided; however, 12 cases of perforation would have been missed. In this study, patients were not sub-grouped according to the presence of immunocompromising. Van de Wall et al. [25] found that the optimal CRP threshold distinguishing complicated from uncomplicated diverticulitis was 175 mg/l, but 39% of patients with a complicated episode had a CRP below this threshold. Thus, a low CRP does not exclude complicated diverticulitis. Focusing on elderly patients, as previously reported, the clinical presentation of ALCD is more nuanced. Only 50% of patients older than 65 years with ALCD have pain in the lower quadrants of the abdomen, with only 17% having fever and 43% do not have a leucocytosis [4].

2. *What is the optimum pathway for imaging in elderly patients with suspected acute left colonic diverticulitis? CT or US or both?*

- **Statement 2.1 We suggest the use of CT-scan with IV-contrast in all elderly patients with suspected diverticulitis to confirm the diagnosis and to distinguish complicated from non-complicated diverticulitis** ***. [Conditional recommendation, very low-quality evidence]***
- **Statement 2.2 In elderly patients with suspected diverticulitis who cannot undergo CT-scan with IV-contrast (i.e. severe acute or chronic kidney disease or contrast allergy), we suggest the use of US, MRI or CT-scan without IV-contrast as alternative diagnostic approaches, according to resources availability.** ***[Conditional recommendation, very low-quality evidence]***

According to the WSES Guidelines [13, 14] in the general population, regarding CT scans of the abdomen and pelvis, this is indicated for all patients with suspected ALCD, but ultrasound (US) may be a useful alternative in the initial evaluation of patients. A step-up approach with CT performed after an inconclusive or negative US may be a safe approach for suspected acute diverticulitis in the general population [13, 14]. US could be used to confirm or exclude other conditions which do not require CT. The reported sensitivity and specificity are 0.95 and 0.96–0.99 for CT scan and 0.90 and 0.90–1.00 for US, respectively [18, 19]. In a meta-analysis by Andeweg et al [19], the pooled specificity of CT (96% [95% CI 90–100%]) was significantly higher compared with US (90% [95% CI 86–94%] (*p* = 0.04; OR 2.46; 95% CI 1.01–5.96) and an accurate diagnosis was made in 68% of patients with a CT scan and in 48% with US (*p* = 0.002, OR 2.6; 95%CI 1.41–4.93). In the study by Nielsen et al [26] in cases of uncomplicated diverticulitis CT scan and US results were not comparable in 17% of cases (no diverticulitis on US in 14% and inconclusive US in 3% of cases) and in cases of complicated diverticulitis they were not comparable in 79% of cases (uncomplicated diverticulitis on US in 34%, no diverticulitis on US in 28% and inconclusive US in 17% of cases). The importance of CT imaging also emerged from the propensity score analysis by Shin et al in which the presence of distant intraperitoneal air on CT was the only significant parameter that correlated with operative management of colonic diverticulitis [27]. Focusing on elderly patients, among the 464 patients older than 80 years presenting to the ED with acute abdominal pain [28], 55% had positive CT scans and 9% had diverticulitis. The clinical diagnosis obtained was clinically unsuspected prior to CT in 43% (*p* < 0.05) with significant difficulty in diagnosing of diverticulitis (*p* < 0.01). Furthermore, CT results influenced treatment plans in 65% overall, surgical in 48% of these and medical in 52%.

Hence, the importance of performing abdominal and pelvic CT scan with IV-contrast in all elderly patients with suspected ALCD (regardless of the value of leukocytes and of CRP) to exclude other diagnoses and to distinguish complicated from non-complicated diverticulitis, and to promptly plan the correct treatment.

The prevalence of chronic kidney disease in the US is 39.4% of persons aged 60+ years versus 12.6 and 8.5% of persons aged 40–59 years and 20–39 years, respectively [29]. However, the high prevalence of kidney disease among elderly patients should not discourage CT scan execution with IV-contrast, because, usually, a prompt diagnosis and treatment in this frail population may justify the risk of contrast-induced acute kidney injury (CI-AKI).

Even if there is general agreement that chronic kidney disease represents the most significant independent predictor of CI-AKI [30], especially in the subset of patients with eGFR ≤ 45 ml/min[31], a recent meta-analysis of retrospective cohort studies of IV radiographic contrast failed to show a higher risk of CI-AKI after CT-scan in patients with chronic kidney disease [32].

This finding is consistent with other recent studies [33–37]. Clinicians should reassess the weight attributed to potential CI-AKI in their decision-making process. Furthermore, in addition to the patient-related risk factors for CI-AKI (advanced age, chronic kidney disease, diabetes mellitus, reduced effective circulating volume, congestive heart failure, anaemia, kidney transplant and concomitant nephrotoxic drugs), modifiable factors related to the procedure may play a role in the occurrence of CI-AKI. These include contrast media volume, route of contrast administration (intra-arterial versus intravenous), type of contrast media, and repeated procedures in a narrow temporal window [30]. Furthermore, the concomitant and crucial resuscitation with crystalloids and antibiotics would minimize the incidence of CI-AKI.

Alternative diagnostic approaches, even if less accurate, in elderly patients with suspected ALCD who cannot undergo CT-scan with IV-contrast exist. They are US, MRI or CT-scan without IV-contrast. The reported sensitivity and specificity of MRI for ALCD are 0.98 and 0.70–0.78, respectively [19]. However, MRI is rarely a feasible imaging modality in an urgent setting. Concerning unenhanced CT, in a study of 208 patients older than 75 years presenting to ED with acute abdominal pain, radiologists changed the diagnosis suspected by the emergency physicians in 59.1% of patients with unenhanced CT images only and in 61.2% of patients with both unenhanced and contrast-enhanced CT. The addition of contrast medium did not significantly improve the frequency of change in diagnosis (*p* = 0.746) [38]. However, the evidence is insufficient to recommend it as the first-choice examination. Furthermore, in a recent prospective study on patients with suspected diverticulitis, although unenhanced low-dose CT showed good sensitivity (98.6%) for the detection of diverticulitis with excellent intermodality agreement, it had significantly lower sensitivity (61%) for the detection of complications [39].

Recently, the use of point-of-care ultrasound (POCUS) by non-radiologists has dramatically increased. It could have a role in the diagnosis of acute diverticulitis and in those elderly patients who cannot undergo CT-scan [40, 41]. Generally, abnormal ultrasound findings in ALCD include a thickened wall of more than 4 mm, non-compressibility and loss of peristalsis. The layers of the colonic wall are usually preserved in diverticulitis compared with malignant tumours. Furthermore, POCUS may detect complications of diverticulitis which may include abscess, free intraperitoneal fluid, and free intraperitoneal air [40].

### Management

3. *What is the best treatment for uncomplicated diverticulitis (WSES stage 0) in elderly patients?*
4. *What is the best treatment for localized complicated diverticulitis without abscess (WSES stage 1a) diverticulitis in elderly patients?*

- **Statement 3.1 We suggest that antibiotic therapy should be avoided in immunocompetent elderly patients with uncomplicated left colonic diverticulitis (WSES stage 0) without sepsis-related organ failures** ***[Conditional recommendation, very low-quality of evidence]***
- **Statement 4.1 We suggest antibiotic therapy administration for elderly patients with localized complicated left colonic diverticulitis with pericolic air bubbles or little pericolic fluid without abscess (WSES stage 1a).** ***[Conditional recommendation, moderate quality of evidence]***

According to the WSES guidelines [13, 14], antibiotic therapy can be avoided in immunocompetent patients with uncomplicated diverticulitis without systemic manifestations of infection. The AVOD trial [42] involved ten surgical departments in Sweden and one in Iceland and recruited 623 patients with a mean age of 57 years with CT-verified uncomplicated ALCD. Patients were randomized to treatment with (314 patients) or without (309 patients) antibiotics. Antibiotic treatment in patients with uncomplicated ALCD (without CT signs of abscess, fistula, or free air) neither accelerates recovery nor prevents complications or recurrences. The DIABOLO trial [43] included 528 patients with CT-proven, primary, left-sided, uncomplicated, ALCD (Hinchey 1a-1b) with age ranging from 48 to 64 years. Amoxicillin plus clavulanic acid 1.2 g four times daily intravenously for at least 48 hours (after which the route was switched to oral administration of 625 mg three times daily), for a first episode of CT-proven uncomplicated ALCD, demonstrated that observational treatment without antibiotics did not prolong recovery and could be considered appropriate in patients with uncomplicated diverticulitis. Even if no significant differences between Hinchey stages 1a and 1b diverticulitis were found, it should be noted that the vast majority of patients included had a diagnosis of Hinchey stage 1a ALCD (90.1% in the observational and 94% in the antibiotic-treated group) with only a small percentage of patients with Hinchey stage 1b diverticulitis.

Omitting antibiotics in the treatment of uncomplicated ALCD did not result in more complicated diverticulitis, recurrent diverticulitis or sigmoid resections at long-term follow-up. As the DIABOLO trial was not powered for these secondary outcome measures, some uncertainty remains whether (small) non-significant differences could be true associations.

A systematic review and meta-analysis including 2469 patients (including elderly patients) with acute uncomplicated diverticulitis [44] showed that treatment of acute uncomplicated diverticulitis without antibiotics might be feasible with outcomes that are comparable to antibiotic treatment and with potential benefits for patients and the overall health economic system.

Furthermore, antibiotic use in patients with acute uncomplicated diverticulitis seems to increase the length of hospital stay [45].

Finally, current evidence [46] does not support the administration of antibiotics to improve outcomes in carefully selected healthy patients with uncomplicated ALCD. However, this evidence comes from young patients (mean age in AVOD trial: 57; age range in DIABOLO trial: 48-64). For this reason, for elderly patients, the recommendation has very low-quality evidence. Further studies should help identify specific subpopulations of patients who would derive benefit from antibiotic therapy and help define appropriate antibiotic regimens and treatment durations that minimize cost, adverse effects, and risk of anti-microbial resistance. Due to the low event rate, it remains uncertain whether antibiotic treatment is necessary in cases with isolated pericolic gas, whether in young or elderly patients [14].

5. *What Is the best treatment for left colonic diverticulitis with abscess (WSES 1b-2a) in elderly patients?*

- **Statement 5.1 In elderly stable patients with an abscess from acute left colonic diverticulitis (WSES stage 1b-2a) and without peritonitis, we suggest the administration of a broad-spectrum antibiotic therapy.** ***[Conditional recommendation, very low quality of evidences]***
- **Statement 5.2 We suggest adding percutaneous drainage to antibiotic therapy in elderly patients with acute left colonic diverticulitis and an abscess larger than 4 cm (WSES stage 2a), when skills and facilities are available. Cultures from percutaneous drainage should be carried out to guide the antibiotic therapy.** ***[Conditional recommendation, very low quality of evidences]***

ALCD is associated with an abscess in nearly 20% of cases [47]. Current evidence is of poor quality with no randomized studies, and there are no studies focused on the elderly.

The rationale for percutaneous drainage is to remove the source of sepsis where antibiotics could fail to reach adequate concentrations, with a consequent failure of the non-operative treatment. As with all the non-operative management strategies, it is indicated only in patients who remain stable without septic shock, where prompt and adequate source control is mandatory.

The treatment with percutaneous drainage has been considered the preferred treatment option since its dissemination and generally, a size of 4 cm has been considered as the cut-off for the indication to drain [48–51].

In a systematic review of observational and retrospective studies, the treatment of abscess with a median size of 4 cm (range: 1.5–5-0 cm) with antibiotic therapy alone failed in 18.7% of cases with a low mortality rate; percutaneous drainage of abscesses with a median size of 6.1 cm (range 4.6–8.7 cm) failed in 21.1% [52]. Surgery is associated with a higher mortality rate in the elderly, and it is not the preferred first-line option in stable patients. Rather, it is reserved for failure of non-operative management.

A retrospective observational study from Finland addressed the treatment of diverticular abscesses larger than 40mm with antibiotics only or with the addition of percutaneous drainage. The study results demonstrated that the outcomes were comparable with similar failure rates (44% for antibiotics and 33% for drainage) and similar morbidity and mortality; however, the study was retrospective and was not focused on the elderly [53]. In elderly patient, we suggest adding percutaneous drainage to antibiotic therapy in cases with an abscess larger than 4 cm. When skills and facilities are not available, we recommend to consider transferring the patient to a higher level hospital.

6. *What is the best treatment for elderly patients with acute diverticulitis with distant free intraperitoneal air and without diffuse fluid (WSES stage 2b)?*

- **Statement 6.1: In elderly patients with acute left colonic diverticulitis and CT findings of distant intraperitoneal free air and no free fluid (WSES stage 2b) we suggest against non-operative management as a viable option.** ***[Conditional recommendation, very low quality of evidences]***

The presence of free abdominal air is generally considered as a surgical indication. The non-operative management in patients with abdominal free air without diffuse fluid and generalized peritonitis has been described in several studies [54–56]. WSES guidelines suggest a non-operative treatment only in selected patients with distant air and without diffuse fluid [14]. Non-operative management with antibiotic therapy was associated with a high failure rate, ranging from 10 to 43%. It should be noted that the studies also included patients with pericolic free air and only a small proportion of patients with distant free air. None of the studies focused on the elderly and were retrospective, generating a very low-level quality of evidence. The available evidence does not support the indication for non-operative management in cases with distant abdominal free air in elderly patients, and surgical exploration is suggested.

7. *What is the best treatment for elderly patients with acute diverticulitis and diffuse peritonitis (WSES stage 3–4)?*

- **Statement 7.1 In elderly patients with acute left colonic diverticulitis and diffuse peritonitis (WSES stage 3–4) we recommend against non-operative management as a viable option.** ***[Strong recommendation, very low quality of evidences]***
- **Statement 7.2 In elderly patients with acute left colonic diverticulitis and diffuse peritonitis (WSES stage 3–4) we recommend prompt and effective source control surgery. [** ***Strong recommendation, very low quality of evidences]***

Patients with diffuse peritonitis are often critically unwell and require immediate fluid resuscitation, antibiotic treatment and surgery. Although perforated diverticulitis complicated by diffuse peritonitis has a low absolute prevalence, it has a high postoperative mortality, regardless of surgical technique [13].

8. *When is a planned elective sigmoid resection indicated in elderly patients with left colonic diverticular disease?*

- **Statement 8.1 We suggest against elective sigmoid resection after a conservatively treated episode of acute left colonic diverticulitis in asymptomatic elderly patients without stenosis, fistulae or recurrent diverticular bleeding.** ***[Conditional recommendation, very low-quality evidence]***
- **Statement 8.2 We suggest considering elective sigmoid resection after a conservatively treated episode of acute left colonic diverticulitis in high-risk elderly patients, such as immunocompromised patients (if fit for surgery).** ***[Conditional recommendation, very low-quality evidence]***
- **Statement 8.3 We suggest elective sigmoid resection in elderly patients (if fit for surgery) with left colonic diverticular disease complicated with stenosis, fistulae or recurrent diverticular bleeding.** ***[Conditional recommendation, very low-quality evidence]***
- **Statement 8.4 We suggest elective sigmoid resection in elderly patients (if fit for surgery) with very symptomatic left colonic diverticular disease, which compromise the quality of life.** ***[Conditional recommendation, very low-quality evidence]***

As reported above, according to most authors [7, 9, 12], the risk of recurrence after a first episode of ALCD in the elderly population is significantly lower than in younger patients. In observational studies [57] of younger patients, the reported risk of recurrent ALCD after a medically treated diverticular abscess was up to 60%. In other studies on patients with a mean age of 60 years, the reported recurrence rate was 9–30% [58, 59], with only 2.7% requiring sigmoid resection with a stoma and no mortality reported [59].

Furthermore, in an article by Lidor et al., the postoperative mortality and morbidity was higher in the elderly population after urgent sigmoid resection for ALCD. However, the effect of age on postoperative mortality was more pronounced after elective surgery, where mortality rates ranged from 0.56% in patients 65–69 years old up to 6.5% in patients older than 85 years old [60]. However, in the article, the causes of mortality were not reported. Furthermore, because many immunocompromised patients are operated on electively after conservative treatment while many immunocompetent patients are not, there can be a bias because more immunocompromised patients may have been operated on in an elective fashion.

As stated in the WSES Guidelines [13, 14], patient-related factors and not the number of previous diverticulitis episodes should be considered in planning elective sigmoid resection in patients with ALCD treated non-operatively.

Thus, taking into account the low risk of recurrence after a first episode of diverticulitis in the elderly population, even in the presence of a medically treated diverticular abscess, with the high postoperative mortality and morbidity after elective surgery for diverticulitis in this population, a case-by-case balance of risks and benefits should be made. If, after a medically resolved episode of ALCD, the diverticular disease is asymptomatic or mildly symptomatic, we suggest against planning elective sigmoid resection. On the other hand, if there is very symptomatic disease or complicated by stenosis, fistulae, or recurrent diverticular bleeding, compromises the patient’s quality of life, an elective sigmoid resection in patients who are fit for surgery could be considered.

Immunocompromised patients need to be evaluated carefully, which includes patients such as those with organ transplant and patients using corticosteroids. Because they are at increased risk of having complicated diverticulitis requiring emergency surgery [61–64], as stated in the WSES Guidelines [13, 14], after a conservatively treated episode of ALCD an elective sigmoid resection should be planned.

9. *Is endoscopic screening recommended for elderly patients treated with non-operative management for acute left colonic diverticulitis?*

- **Statement 9.1 We suggest planning early colonic evaluation in elderly patients after an episode of acute left colonic diverticulitis. [Conditional recommendation, very low-quality evidence]**

A recent large retrospective study [65] on 932,860 patients with the first episode of diverticulitis showed that individuals are twice as likely to be diagnosed with colorectal cancer within one year of their first episode of acute diverticulitis compared with individuals without diverticulitis. The WSES guidelines [13, 14] recommended that endoscopic evaluation be performed after an episode of complicated ALCD to rule out colorectal cancer. However, the indication for colonoscopy is debated in patients with uncomplicated episodes. In a systematic review and meta-analysis performed by Meyer et al., they found a pooled prevalence of colorectal cancer of 1.3% (95% CI 0.1–2.0%) among 3834 patients with uncomplicated episodes [66, 67]. Similarly, a recent publication reported colorectal cancer prevalence to be 1.8% in a cohort of 227 patients with uncomplicated ALCD [68]. Furthermore, age older than 50 years was found to be a significant risk factor for advanced colonic neoplasia at follow-up colonoscopy after medically treated acute uncomplicated ALCD [69].

With this in mind, we suggest that in elderly patients, an early colonic evaluation (colonoscopy or CT colonography) after a conservatively treated episode of ALCD should be considered.

### Surgical technique

10. *Should laparoscopic peritoneal lavage and drainage be considered in elderly patients with acute diverticulitis?*

- **Statement 10.1 In elderly patients with acute left colonic diverticulitis and acute peritonitis we suggest against laparoscopic lavage as the preferred surgical approach due to the higher risk of failure to control the source of sepsis.** ***[Conditional recommendation, moderate quality evidences]***

In case of diffuse purulent peritonitis (Hinchey III) laparoscopic peritoneal lavage has been proposed as alternative to colonic resection. Three randomized trials have been published addressing this topic, and several meta-analyses summarized their findings, focusing the interest for this potentially less invasive approach among the scientific community [70–78]. This minimally invasive approach with laparoscopic lavage has been demonstrated to have similar short-term mortality and similar long-term results [79]. However, patients treated with laparoscopic lavage had a considerably higher incidence of intra-abdominal abscesses and higher reoperation rates during the index admission due to inadequate source control. The mentioned studies did not include elderly patients, and therefore, the results cannot be easily generalized. Source control, in elderly and frail patients, must be the most important target in the treatment of ALCD, avoiding the risk of a second “septic hit” derived from incomplete treatment. Therefore, we suggest against laparoscopic lavage as the preferred technique in managing elderly patients with Hinchey III ALCD. This option could be considered in very selected, well-informed patients after considering the risks and the potential benefit of a lesser invasive procedure.

11. *What is the best surgical procedure for elderly patients with perforated diverticulitis with generalized peritonitis: Hartmann resection or resection with primary anastomosis or damage control surgery?*

- **Statement 11.1 We suggest that in elderly patients with perforated diverticulitis with generalized peritonitis, Hartmann operation and resection with primary anastomosis are both reasonable options.** ***[Conditional recommendation, low-quality of evidence]***
- **Statement 11.2 We suggest that in elderly patients with perforated diverticulitis with generalized peritonitis and physiological derangement, Damage Control Surgery (emergency laparotomy, source control and application of open abdomen and abdominal vacuum-assisted closure) may be a viable option. [Conditional recommendation, very low quality of evidence]**

According to the WSES guidelines [13, 14], the Hartmann technique is still recommended for patients with diffuse peritonitis who are critically unwell or have numerous comorbidities. Primary resection with anastomosis with or without a diverting stoma may be performed in clinically stable patients with no comorbidities. For clinically unstable patients with diverticular peritonitis (severe sepsis/septic shock), a damage control surgery technique may be recommended.

Despite limitations due to the lack of strong evidence, some authors [80, 81] have attempted to propose a treatment strategy for various clinical scenarios of ALCD in the general population.

A meta-analysis by Halim et al. [82] including 3,546 patients with Hinchey III and IV diverticulitis comparing Hartmann procedure (HP) and resection and primary anastomosis (R-PA) with or without faecal diversion, included 22 observational studies and 3 RCTs. The overall mortality in the HP group was 10.8% across the observational studies and 9.4% in the RCTs. The mortality rates in the R-PA group, 8.2% in the observational studies and 4.3% in the RCTs, were lower than those in the HP group. They found a 40% lower mortality rate in the R-PA group than in the HP (OR 0.60, 95% CI 0.38–0.95, *p* = 0.03) when analysing the observational studies. However, meta-analysis of the three RCTs did not demonstrate any difference in mortality.

Another recent meta-analysis [83] found that R-PA was associated with better short- and long-term outcomes at the cost of significantly longer operating time at emergency surgery. No subgroup analyses based on age were made, nor specific studies on elderly patients.

A recent large retrospective study [84] on 10780 urgent/emergent colectomies for patients with diverticulitis showed that postoperative mortality was twofold greater when non-colorectal surgeons performed R-PA vs HP (15% vs 7.4%; *p* < 0.001) and 1.4 times greater among non-colorectal surgeons than among colorectal surgeons (7.5% vs 5.3%; *p* = 0.04). On multivariable logistic regression (adjusting for patient demographics/characteristics, year, hospital academic status and surgeon training) R-PA remained associated with increased mortality (OR 2.7; 95% CI 1.7–4.4; *p* < 0.001), complications (OR 1.8; 95% CI 1.3–2.5; *p* < 0.001), and reoperation (OR 3.4; 95% CI 1.8–6.3; *p* < 0.001). However, the patients included within this study were not randomly assigned to each treatment arm. Then, patients who were selected to receive a R-PA may be assumed to have been less ill on presentation.

The recent WSES guidelines covering the management of intra-abdominal sepsis [85] stated that there is insufficient evidence to advocate for damage control surgery (DCS) as a general strategy in patients with secondary peritonitis. On the other hand, even though it comes with a low grade of recommendation, the same guidelines suggest that DCS may be an option in selected significantly physiologically deranged patients with ongoing sepsis. There are few case series about DCS emergency laparotomy with the temporary application of abdominal vacuum-assisted closure (VAC) for perforated diverticulitis, including also elderly patients [86–90]. The Perathoner et al. [87] series included 15 patients (median age 68 years, range 35–89) who underwent DCS with lavage, limited resection of the diseased colonic segment, abdominal VAC, and second-look operation. Of these, nine had intestinal continuity restored during a second-look operation, with one patient developing anastomotic leakage. Sohn et al. [86] reported 58 patients (median age 70 years, range 30–92) who underwent DCS for Hinchey III (81%) and Hinchey IV (19%) ALCD, reporting a secondary bowel restoration rate of 83% with a 24% of loop ileostomy rate. Overall, 10% of patients experienced an anastomotic leak that eventually required the creation of a stoma: at the end of the hospital stay, they documented a 50% rate of patients without a stoma. DCS led to a significantly reduced stoma rate after the initial hospital stay without an increased risk of postoperative morbidity [91].The study by Kafka-Ritsh et al. [88] included 51 patients (median age 69 years, range 28–87) with diverticular peritonitis treated with DCS with a rate of bowel restoration at the second-look operation of 76% and with a 11% rate of loop ileostomy. Their fistula rate was 13%. Tartaglia et al. reported 34 patients (13 Hinchey III and 21 Hinchey IV) with a mean age of 66.9 years and a Mannheim Peritonitis Index (MPI) of 25.12 (SD ± 6.28) treated with DCS: 24 patients (71%) had restoration of bowel continuity, 10 (29%) patients had an end colostomy (HP) and 1 patient had an anastomotic leak. The mortality rate was 12%.

A recent retrospective study [92], including 290 patients who underwent urgent laparotomy for non-trauma peritonitis, compared DCS and definitive initial surgical management. They found that DCS in severe non-trauma peritonitis patients was feasible and safe as surgical strategy management without increasing mortality, length hospital of stay, stoma rate or complications.

There are insufficient data focused on elderly patients to recommend or suggest R-PA instead of HP. Both are reasonable options in cases of perforated diverticulitis with generalized peritonitis. The choice could be based on the patient's current clinical status and pre-existing conditions (degree of autonomy, comorbidity, medication taken). DCS could be a viable option in cases with severe physiological derangement.

12. *Should emergency laparoscopic sigmoidectomy (ELS) be considered in elderly patients with perforated diverticulitis with diffuse peritonitis?*

- **Statement 12.1 We suggest that in stable elderly patients with perforated diverticulitis with diffuse peritonitis emergency laparoscopic sigmoidectomy can be performed by experienced laparoscopic surgeons** ***[Conditional recommendation, very low quality of evidence]***

Laparoscopic sigmoidectomy for diverticulitis was initially confined to the elective setting. However, in stable patients and if performed by experienced hands, ELS may also be feasible in Hinchey III and IV ALCD with reported good outcomes [93–95]. In a propensity score-matched cohort of patients with perforated diverticulitis [96], ELS was superior to open sigmoidectomy with regard to postoperative morbidity and hospital stay. However, these results should be interpreted with caution as the cohort consists of selected patients with more favourable characteristics and young age (mean age 56 years), and surgery was performed by experienced gastrointestinal surgeons.

### Antibiotic therapy

13. *What is the best anti-microbial regimen for elderly patients with localized complicated diverticulitis?*
14. *What is the best anti-microbial regimen for elderly patients with perforated diverticulitis with diffuse peritonitis?*

- **Statement 13.1 In elderly patients with localized complicated diverticulitis the empirically designed anti-microbial regimen depends on the underlying clinical condition of the patient, the pathogens presumed to be involved, and the risk factors indicative of major resistance patterns.** ***[Conditional recommendation, very low quality of evidence]***
- **Statement 14.1 In elderly patients with perforated diverticulitis with diffuse peritonitis the empirically designed anti-microbial regimen depends on the underlying clinical condition of the patient, the pathogens presumed to be involved, and the risk factors indicative of major resistance patterns. [Conditional recommendation, very low quality per indirectness]**

According to the WSES guidelines [12, 14], the empirically planned antimicrobial regimen is based on the patient's underlying clinical condition, the microorganisms suspected of being involved, and risk factors for significant resistance patterns.﻿ The principles of empiric antibiotic treatment should be developed based on the most commonly isolated microorganisms, while also taking into account the local trend of antibiotic resistance [85]. The presence of anaerobes and Gram-negative bacteria in the lower gastrointestinal tract should be taken into account while choosing empirical therapy for acute diverticulitis. In addition, quinolone and carbapenem resistance, as well as the presence of ESBL-producing bacteria in the local environment and the location of recent travel, should always be taken into account.

Because inoculation in a healthcare facility, corticosteroid usage, organ transplantation, baseline pulmonary or hepatic disease, and past anti-microbial therapy are the most relevant factors in predicting the presence of resistant bacteria, elderly individuals frequently fall into this category. One of the factors that is significantly linked to poor outcomes in critically ill patients is an ineffective or inadequate antimicrobial treatment. In patients with organ dysfunction and septic shock, broad empiric antimicrobial therapy should be started as soon as possible [97].

Furthermore, for individuals at risk for resistant bacteria, intraperitoneal samples for microbiological evaluation are always indicated from the site of infection. When the findings of microbiological tests are available, the patient should be reviewed, and antimicrobial de-escalation or withdrawal should be considered [98].

15. *When should discontinuation of anti-microbial treatment be considered?*

- **Statement 15.1 In elderly patients with complicated diverticulitis a short course of antibiotic therapy (3–5 days) after adequate source control is a reasonable option.** ***[Conditional recommendation, moderate quality of evidence]***
- **Statement 15.2 In elderly patients with complicated diverticulitis who have ongoing signs of peritonitis or systemic illness (ongoing infection) beyond 5 to 7 days of antibiotic treatment, further diagnostic investigation is indicated. [** ***Conditional recommendation, low quality of evidence*** **]**

The outcomes after 4 days of antibiotic therapy were similar to those following a longer course of antibiotics in patients with intraabdominal infections who completed an acceptable source control regimen [99, 100].

Although discontinuation of anti-microbial treatment should be based on clinical criteria, a 4–6 day period of postoperative anti-microbial therapy in complicated ALCD is suggested if source control has been adequate [12]. Patients who have persistent symptoms of peritonitis or systemic sickness (ongoing infection) after 5 to 7 days of antibiotic treatment should be re-evaluated.

## Conclusion

After the publication of the WSES guidelines for the management of acute left-sided colonic diverticulitis in the emergency setting [13, 14] for the diagnosis and management of ALCD in the general population, the present guidelines represent, to the best of our knowledge, the first clinical guidelines for diagnosis and management of ALCD in elderly patients (> 65 years of age).

**Fig. 2:**
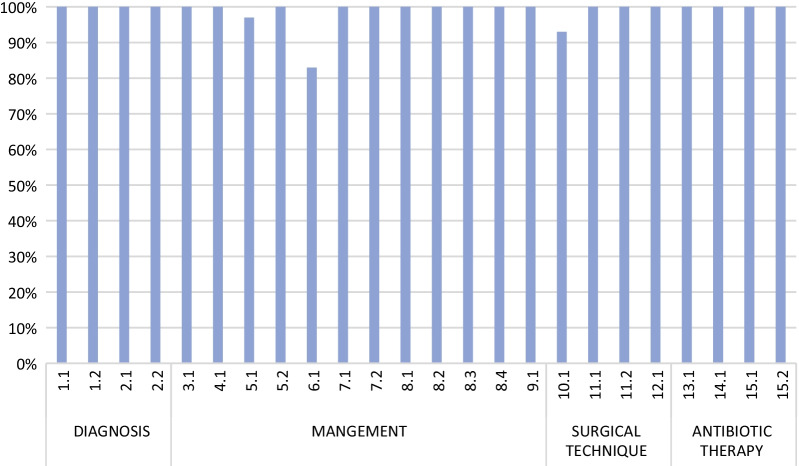
Voting results

During the 1° Pisa Workshop of Acute Care & Trauma Surgery held in Pisa (Italy) in September 2019 with the collaboration of WSES, SICG, ACOI, SICUT, AcEMC and SIFIPAC, a panel of experts participated in a Consensus Conference where three panel-members presented a number of statements, which were developed for each of the four topics regarding the diagnosis and management of ALCD in elderly patients, formulated according to the GRADE system: Diagnosis, Management, Surgical Technique, Antibiotic Therapy. The statements were then voted (Fig. 2), eventually modified and finally approved by the participants to The Consensus Conference (Table 1).

**Table 1:** Summary of recommendations

*Diagnosis*
Statement 1.1 In the elderly population, we suggest against basing the diagnosis of acute left colonic diverticulitis on only patient clinical signs, symptoms and laboratory tests. *[Conditional recommendation, very low-quality evidence]*
Statement 1.2 We suggest that elderly patients presenting with abdominal guarding or pain in the lower left abdomen on physical examination undergo appropriate imaging for suspected diverticulitis, regardless of the value of leukocytes and of C-reactive protein (CRP). *[Conditional recommendation, very low-quality evidence]*
Statement 2.1 We suggest the use of CT-scan with IV-contrast in all elderly patients with suspected diverticulitis to confirm the diagnosis and to distinguish complicated from non-complicated diverticulitis*. [Conditional recommendation, very low-quality evidence]*
Statement 2.2 In elderly patients with suspected diverticulitis who cannot undergo CT-scan with IV-contrast (i.e. severe acute or chronic kidney disease or contrast allergy), we suggest the use of US, MRI or CT-scan without IV-contrast as alternative diagnostic approaches, according to resources availability. *[Conditional recommendation, very low-quality evidence]*
*Management*
Statement 3.1 We suggest that antibiotic therapy should be avoided in immunocompetent elderly patients with uncomplicated left colonic diverticulitis (WSES stage 0) without sepsis-related organ failures *[Conditional recommendation, very low-quality of evidence]*
Statement 4.1 We suggest antibiotic therapy administration for elderly patients with localized complicated left colonic diverticulitis with pericolic air bubbles or little pericolic fluid without abscess (WSES stage 1a). *[Conditional recommendation, moderate quality of evidence]*
Statement 5.1 In elderly stable patients with an abscess from acute left colonic diverticulitis (WSES stage 1b-2a) and without peritonitis, we suggest the administration of a broad-spectrum antibiotic therapy. *[Conditional recommendation, very low quality of evidences]*
Statement 5.2 We suggest adding percutaneous drainage to antibiotic therapy in elderly patients with acute left colonic diverticulitis and an abscess larger than 4 cm (WSES stage 2a), when skills and facilities are available. Cultures from percutaneous drainage should be carried out to guide the antibiotic therapy. *[Conditional recommendation, very low quality of evidences]*
Statement 6.1: In elderly patients with acute left colonic diverticulitis and CT findings of distant intraperitoneal free air and no free fluid (WSES stage 2b), we suggest against non-operative management as a viable option. *[Conditional recommendation, very low quality of evidences]*
Statement 7.1 In elderly patients with acute left colonic diverticulitis and diffuse peritonitis (WSES stage 3–4), we recommend against non-operative management as a viable option. *[Strong recommendation, very low quality of evidences]*
Statement 7.2 In elderly patients with acute left colonic diverticulitis and diffuse peritonitis (WSES stage 3–4), we recommend prompt and effective source control surgery. [*Strong recommendation, very low quality of evidences]*
Statement 8.1 We suggest against elective sigmoid resection after a conservatively treated episode of acute left colonic diverticulitis in asymptomatic elderly patients without stenosis, fistulae or recurrent diverticular bleeding. *[Conditional recommendation, very low-quality evidence]*
Statement 8.2 We suggest to consider elective sigmoid resection after a conservatively treated episode of acute left colonic diverticulitis in high-risk elderly patients, such as immunocompromised patients (if fit for surgery). *[Conditional recommendation, very-low quality evidence]*
Statement 8.3 We suggest elective sigmoid resection in elderly patients (if fit for surgery) with left colonic diverticular disease complicated with stenosis, fistulae or recurrent diverticular bleeding. *[Conditional recommendation, very low-quality evidence]*
Statement 8.4 We suggest elective sigmoid resection in elderly patients (if fit for surgery) with very symptomatic left colonic diverticular disease which compromise the quality of life. *[Conditional recommendation, very low-quality evidence]*
Statement 9.1 We suggest planning early colonic evaluation in elderly patients after an episode of acute left colonic diverticulitis. [Conditional recommendation, very low-quality evidence]
*Surgical technique*
Statement 10.1 In elderly patients with acute left colonic diverticulitis and acute peritonitis, we suggest against laparoscopic lavage as the preferred surgical approach due to the higher risk of failure to control the source of sepsis. *[Conditional recommendation, moderate quality evidences]*
Statement 11.1 We suggest that in elderly patients with perforated diverticulitis with generalized peritonitis Hartmann operation and resection with primary anastomosis are both reasonable options. *[Conditional recommendation, low-quality of evidence]*
Statement 11.2 We suggest that in elderly patients with perforated diverticulitis with generalized peritonitis and physiological derangement, Damage Control Surgery (emergency laparotomy, source control and application of open abdomen and abdominal vacuum-assisted closure) may be a viable option. [Conditional recommendation, very low quality of evidence]
Statement 12.1 We suggest that in stable elderly patients with perforated diverticulitis with diffuse peritonitis emergency laparoscopic sigmoidectomy can be performed by experienced laparoscopic surgeons *[Conditional recommendation, very low quality of evidence]*
*Antibiotic therapy*
Statement 13.1 In elderly patients with localized complicated diverticulitis the empirically designed anti-microbial regimen depends on the underlying clinical condition of the patient, the pathogens presumed to be involved, and the risk factors indicative of major resistance patterns. *[Conditional recommendation, very low quality of evidence]*
Statement 14.1 In elderly patients with perforated diverticulitis with diffuse peritonitis the empirically designed anti-microbial regimen depends on the underlying clinical condition of the patient, the pathogens presumed to be involved, and the risk factors indicative of major resistance patterns. [Conditional recommendation, very low quality per indirectness]
Statement 15.1 In elderly patients with complicated diverticulitis a short course of antibiotic therapy (3–5 days) after adequate source control is a reasonable option. *[Conditional recommendation, moderate quality of evidence]*
Statement 15.2 In elderly patients with complicated diverticulitis who have ongoing signs of peritonitis or systemic illness (ongoing infection) beyond 5 to 7 days of antibiotic treatment, further diagnostic investigation is indicated. [*Conditional recommendation, low quality of evidence*]

**Fig. 3:**
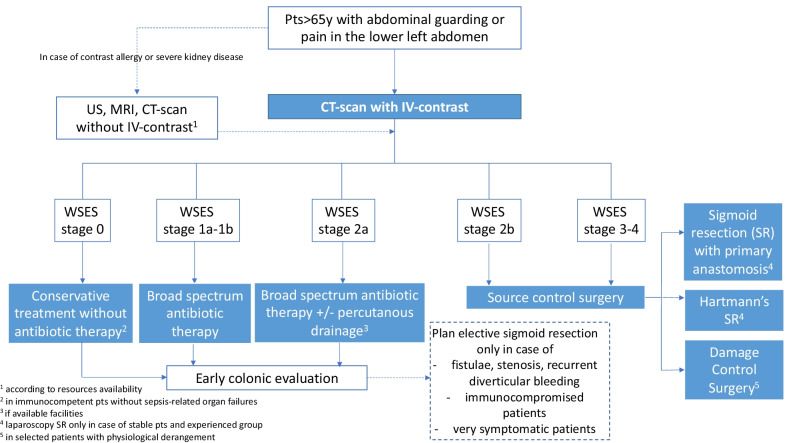
Management algorithm for patients older than 65 years with suspected AD

After reaching consensus on each of the above-mentioned statements, the Scientific Committee members developed the following algorithm for the diagnosis and management of ALCD in Elderly Patient, reported in Fig. 3.

Unfortunately, due to the lack of high-quality studies focusing on elderly patients and to the heterogeneity of the existing studies in the definition of an age cut-off for the characterization of elderly patient, according to the GRADE methodology, all statements are based on low- or very low-quality evidence.

## Data Availability

Not applicable.

## Abbreviations

ALCD: Acute left colonic diverticulitis
WSES: World Society of Emergency Surgery
SICG: Italian Society of Geriatric Surgery
ACOI: Italian Hospital Surgeons Association
SICUT: Italian Emergency Surgery and Trauma Association
AcEMC: Academy of Emergency Medicine and Care
SIFIPAC: Italian Society of Surgical Pathophysiology
SUDD: Symptomatic uncomplicated diverticular disease
NPV: Negative predictive value
ED: Emergency department
CT: Computed tomography
US: Ultrasound
CI-AKI: Contrast-induced acute kidney injury
MRI: Magnetic resonance imaging
IV: Intravenous
POCUS: Point-of-care ultrasound
HR: Hartmann procedure
R-PA: Resection and primary anastomosis
RCT: Randomized controlled trial
DCS: Damage control surgery
VAC: Vacuum-assisted closure
ELS: Emergency laparoscopic sigmoidectomy
